# Framing rights and responsibilities: accounts of women with a history of AIDS activism

**DOI:** 10.1186/1472-698X-11-S3-S7

**Published:** 2011-12-16

**Authors:** Hayley MacGregor, Elizabeth Mills

**Affiliations:** 1 Institute of Development Studies, University of Sussex, Brighton BN1 9RE, UK; 2 AIDS and Society Research Unit, Centre for Social Science Research, University of Cape Town, Cape Town, South Africa

## Abstract

**Background:**

In South Africa, policy with respect to HIV/AIDS has had a strong rights-based framing in line with international trends and in keeping with the constitutional overhaul in the post-Apartheid era. There have also been considerable advances since 1994 towards legal enshrinement of sexual and reproductive health rights and in the provision of related services. Since HIV in this setting has heavily affected women of reproductive age, there has been discussion about the particular needs of this subgroup, especially in the context of service integration. This paper is concerned with the way in which HIV positive women conceptualise these rights and whether they wish and are able to actualise them in their daily lives.

**Methods:**

In 2003 a group of women involved with the Treatment Action Campaign and Medicines Sans Frontières participated in an initiative to ‘map’ their bodies as affected by the virus. A book containing the maps and narratives was published and used as a political tool to pressure the government of the day to roll out antiretroviral therapy (ART) to the population. In 2008, the authors coordinated an initiative that involved conducting follow-up in-depth interviews in which five of these women reflected on those body maps and on how their lives had changed in the intervening five years since gaining the right to treatment through the public sector.

**Results:**

Drawing upon this qualitative data and published sources, these new accounts are analysed in order to reflect the perspectives of these women living with chronic HIV with respect to their sexual relations and fertility desires. The paper reveals difficulties faced by these women in negotiating sexual relationships and disclosure of their HIV positive status. It focuses on how they perceive relative responsibilities in terms of taking preventative measures in sexual encounters. Women adopt tactics within a context characterised by various inequalities in order to ‘make do’, such as by remaining silent about their status. Concerns about childbearing can be addressed by information and support from a health care worker.

**Conclusions:**

Women’s experience of HIV as a chronic illness and the need to adhere to ART, is linked to the way in which the language of responsibility can come to counter-balance a language of rights in treatment programmes.

## Background

In South Africa, the constitution adopted by the democratic regime after 1994 has a prominent focus on individual rights. In the arena of sexual and reproductive health, this has made possible the introduction of a raft of policies that form cornerstones of the provision of basic services and the progressive realisation of enshrined Sexual and Reproductive Health (SRH) rights, in line with international frameworks. Significant progress can be seen, for example, in the introduction of free services in Maternal and Child Health in the public sector, and provision for legal means to terminate pregnancy [1]. However, Cooper et al [1] caution that considerable challenges remain in terms of implementation of policies and provision of new services, particularly in the area of HIV and AIDS. Furthermore, persistent structural factors, such as gender and socio-economic inequalities, continue to impact upon the health status of women in particular. Women have also been most affected by the HIV epidemic that has been a prominent feature of the post-Apartheid era. Amongst women of reproductive age (15-49) in South Africa, 20.2% are estimated to be infected with HIV. The equivalent figure for men is 16.6% [2].

There is a sustained interest evident in published literature in the role that access to SRH services can play in combating HIV infection, and the importance and challenges of integrating SRH services and HIV programmes [3-5]. In particular, the desires, responsibilities and rights of HIV-infected individuals regarding their sexual and reproductive health are singled out for careful consideration [6,7]. Two domains remain particularly contested. These are the sexual activity and fertility of HIV-infected individuals. These issues raise controversial questions regarding the balance between individual rights and public health goals. UNAIDS guidelines [8] underscore the rights of HIV-positive people to a safe and satisfying sex life and are explicitly opposed, for example, to the enactment of criminal legislation regarding the transmission of HIV. These guidelines, as well as those of the World Bank [9], also emphasise autonomy of choice with respect to the ability of HIV-positive people to make informed, free decisions regarding their reproductive capacity, in the absence of discrimination. Human rights directives have been prominent in South Africa from the outset of HIV programming and such principles were already evident in an AIDS Plan developed in a participatory process in the early 1990s, in the run-up to the election of the ANC government [10]. The current South African legal position with respect to these issues is in line with the international recommendations.

Yet disjuncture exists between legal formulations of sexual and reproductive health rights and the realities of life for HIV-positive women in South Africa, and indeed elsewhere. This paper analyses the dilemmas that can arise in a rights-based approach to HIV and SRH with respect to the experience of HIV-positive women in South Africa. It examines how five HIV-positive women, who have a history of AIDS activism and have received several years of HIV care in clinic programmes, have come to understand their rights and responsibilities with respect to their sexual relationships and fertility desires. What rights have practical significance in their everyday lives? This question has to be considered in the light of the fact that they live in an area of the Cape Town Metropolis where, as in similar low-income settlements, a high prevalence of HIV coexists alongside a matrix of inequalities that can shape women’s lives across race, class, sexuality and gender [11,12]. How do they negotiate the compromises that become necessary in their sexual and reproductive lives in a setting where pervasive poverty affects broader wellbeing?

First, we argue that the tension between enshrined sexual and reproductive health rights and lived reality reflects the fact that the international framing of human rights and the constitutional framing of gender equality in South Africa are based on presumptions of sameness. Yet this is a socio-economic context of entrenched disparities that contribute to individuals having different perspectives, beliefs and desires. Gender equality within the liberal model of rights presumes “equality on the grounds of sameness between women and men and between women” ([13]. p4). In South Africa, the lived experience between women, and between women and men, reflects wide-ranging differences in access to education, employment, health care, housing and so forth. Socio-economic inequalities continue to shape lives along the lines of race, closely but not exclusively linked to class [14]. Gender intersects with class and race, and this dynamic is particularly salient in the correlation between gender-based violence, poverty and high rates of HIV-infection amongst women [15-18] living in peri-urban areas in South Africa. Differential knowledge of and access to an over-burdened justice system also undermines the realisation of rights for women who have, for example, been raped and have a right to post-exposure prophylaxis but who are not aware of this right or are unable to insist on its implementation [19]. Similarly, HIV-positive women who wish to have children, and who have a right to access PMTCT (prevention of mother to child transmission), may not know of their rights or may be deterred from enacting these rights by incorrect information or discriminatory health care professionals.

International human rights discourse, and South Africa’s progressive constitution, construct equality through sameness [20], thereby assuming a “unified/universal political subject” ([13]. p5). Anthropological critiques suggest that it is necessary to accord greater understanding to the ways that agency is constrained, thus limiting the extent to which rights can be claimed [21-23]. This paper indicates the value of understanding the textures of HIV-positive women’s lives and the extent to which their sexual and reproductive health rights are circumscribed by the context in which they live and love. Structural violence for poor, black women extends beyond inequalities linked to race, gender and class to include the consequences of these inequalities in women’s alienation from systems that can realise rights, like the justice and medical system. Given these constraints, and in line with de Certeau’s conceptualisation of appropriation and subversion within structural constraints, we suggest that women have developed tactics for ‘making do’ [24].

Second, there are medical ramifications linked to unprotected sex in HIV concordant and discordant couples, for example re-infection by new or resistant viral strains and horizontal transmission. The rights, therefore, of both HIV-negative and HIV-positive people to protection from transmission and re-infection are juxtaposed with HIV-positive women’s legal right to bear children, highlighting the complexities of legal provisions in this context. In South Africa, as elsewhere, the matters of sexual activity and childbearing amongst those infected with HIV attract powerful moralising and social prescriptions [25-27], often shared by health professionals and communities [28,29]. On the positive side, the risks associated with pregnancy, such as peri-natal transmission or disease progression in the mother, are now thought to be substantially reduced by PMTCT and HAART (highly active antiretroviral therapy) [30]. The risk of transmission and re-infection are also significantly reduced when people are receiving ART (antiretroviral therapy), and have an undetectable HIV viral load. In well-resourced settings, artificial insemination and techniques such as sperm washing have also contributed to increasing the reproductive choices of HIV positive persons, but realities in parts of the world where the epidemic remains a significant burden make these measures inaccessible to the majority of those who could benefit.

Even in the absence of legal prescriptions, international guidelines speak of an ethical duty [9] on the part of HIV-positive people to conduct their sexual and reproductive lives in a manner that accepts a responsibility to protect a partner or unborn child. As the UNAIDS guidelines put it, “[p]eople living with HIV, like all people who know or suspect that they are HIV-positive, have a responsibility to practise abstinence or safer sex in order not to expose others to infection” ([8]. p89). A language of responsibility is also reflected in ART and PMTCT programmes: responsibility to others, responsibility to adhere to treatment regimens; and responsibility for maintaining one’s own health as an ‘expert patient’. In conclusion, we consider the manner in which such framings of responsibility can place further burdens upon women who have limited agency and control over their lives. The experience of HIV as a chronic illness and the need to adhere to ART, is linked to the way in which the language of responsibility can come to counter-balance a language of rights in treatment programmes.

## Methods

In 2003 a book, ‘Long Life’ [31], was published, containing the Bambanani Women’s body maps and narratives. This group of women was living with HIV in Khayelitsha, Cape Town, an area previously designated as ‘African’ under the Apartheid regime. Given the emphasis on long life, facilitated by access to ART, Nondusimo Hlwele and the authors of this article came together in 2008 to develop a methodology to investigate how their lives had shifted over five years of living with HIV as a chronic illness. Thereafter, Nondusimo worked with a small subset of women from the original Bambanani Women’s Group who agreed to share their experiences. Signed consent was given by each participant at the outset following full information regarding the aim of the study, the materials that would be collected through participation and the outcomes of the research process, including publications. All the women disclosed their first names along with their status in the Long Life book and have agreed for their body maps and updated accounts to be made publicly available. For ethical purposes we have assigned pseudonyms to each of the participants and have removed all other possible identifying features from the accounts except when express permission was given.

In this paper, we analyse five new accounts from the following women: Nonkosi; Thokosizwe; Busisiwe; Thandiswa and Nandipha. Three sets of information were collected in 2008: individual written narratives; semi-structured in-depth interviews; and photographs with annotations written by each woman. In the case of Nandipha, who had recently died, the follow-up consisted of collecting detailed written accounts from two of the women who were her close friends. Funding was obtained from the Museum of Anthropology and Archaeology at the University of Cambridge which exhibited the body maps of these five women together with the new material. This paper considers the women’s original accounts in the 2003 book alongside the new data. Given the small number of women, we do not claim that the observations are generalisable. However, the dilemmas that these women articulate with respect to their sexual relationships and reproductive options provide an interesting perspective and a point of reference for a reflection upon debates about the rights, responsibilities and specific needs of HIV positive women in these two areas.

## Findings

In 2002-3, when the accounts for the book ‘Long Life’ were being collected, the five women in question ranged in age between twenty-two and thirty-one years. Thandiswa summed up the similarities in their life experience: *“[m]y story is similar to the other women in this book. Get pregnant young*, *don’t finish school*, *find out we are positive*, *lucky to find work*, *like that.” *[31] [p65]. They had all been diagnosed HIV-positive within the previous two years, two of them at routine antenatal visits and the others at the suggestion of medical professionals when their health deteriorated. The latter three were already on ART, the positive effects of which were described in miraculous terms [32]. They were fortunate enough to obtain these life-giving drugs, not at that time available to the general population, at the Médicins sans Frontières (MSF) clinic in Khayelitsha. The book had a strong political rationale: to add the ‘voices’ of ordinary people to the campaign to force the state to ‘roll out’ universal access to ART in the public sector. None of these women had a history of activist involvement, but were recruited for the book initiative through attendance at MSF support groups. The women were thus drawn into a wider activist world involving organisations such as the Treatment Action Campaign (TAC), which were claiming entitlements to healthcare under the auspices of constitutional rights. At the conclusion of the book project they then received training through TAC to become ‘expert’ peer educators, and for a period of several years earned incomes doing HIV-related work. As a group they were thus well exposed to an international language of rights as pertaining to HIV that constituted the foundations of such organisations. Unfortunately, due to a variety of factors, the various pieces of HIV-related work ceased at different points in time for almost all the women. By the time of the follow-up interviews in 2008, only one of the women, Nonkosi, remained in full-time employment in a new but still HIV-related job.

### Responding to a diagnosis of HIV

In their accounts in the book, the women reflected upon how they might have contracted HIV. Busisiwe recounted her relationship history in terms that emphasised a paucity of options. As a child in the rural Transkei she was shunted between the home of her stepfather and her grandmother, fending off the sexual advances of the former. When she entered high school she started seeing a boyfriend. Consequently her stepfather discontinued payment of her school fees. Her boyfriend gave her the money as he was working, and after a while she moved in with him. As her relationship with her stepfather had deteriorated, she felt she had no choice; there was nowhere else to go. This partner openly maintained concurrent sexual partners and thus they fought frequently. She finally left him and met her husband; by the age of twenty she was pregnant and then diagnosed with HIV. It is clear from her account that she assumed her husband to be negative at this time. She knew that her first boyfriend had become very thin and she linked his behaviour to her own infection. She had known of HIV but had not thought that there were people in South Africa with the condition.

Thokosizwe’s account focused heavily upon her abduction and rape by a group of men at the age of twenty. She was unsure as to whether she had contracted HIV from that encounter or from her most recent boyfriend. Nandipha remembered that she could not comprehend at the antenatal clinic how she could be HIV-positive, as she only had one boyfriend at a given time. The other women were also unmarried and linked their HIV infection back to a particular partner. In their assessments the predominant sense is of themselves as victims, let down by men to whom they had been faithful. Nonkosi alone reflected upon her own agency in contracting the virus. The man she suspected had passed on the virus was now very ill. She was unsure if he had infected her purposefully but felt she could not blame him, as she had not used a condom.

What is striking in these accounts in the book is the difficulty that these women experienced with disclosure upon learning of their status. Nandipha did not tell the father of her unborn child as she was concerned that he would leave her, even though she believed he ‘gave’ her the virus. Thokosizwe’s partner seemed to respond supportively to her news and agreed to go for testing himself; but she did not hear from him again and only saw him once more, when he was dying of AIDS in hospital. Busisiwe approached the issue by suggesting that both she and her husband should go for testing. He refused outright and warned her not to tell him her result if she tested, as he could not sleep with her if he knew she was positive. She remained silent and torn by her dilemma: he refused condoms and she felt she could not insist without disclosing; yet she risked giving him the virus. Nonkosi recounted that she had told a man who was showing interest in her of her status and that was the last she saw of him. Thandiswa was also single and joked that she told men of her status to get rid of them when they were giving her unwanted attention. However she commented wryly that it would not be amusing if she actually liked someone. In a discussion reported in the book, the women reflected upon the fact that men seemed unwilling to test but preferred to blame their female partners. They were sympathetic towards Busisiwe’s choice of silence, as the consequences of disclosure could be significant: abandonment, loss of financial support and indeed the very real risk of violence. They were also afraid of the stigma of becoming known as HIV-positive. Nandipha recounted that when she shared with a clinic counsellor her anxiety that her newborn might be positive, she was accused of causing the predicament by ‘playing with her body’. Thokosizwe noted that she heard people pointing and commenting that a woman with HIV must have been behaving ‘like a prostitute’. Indeed, she passed this judgement on her own mother who had died of AIDS, describing her as a woman who did not behave ‘responsibly’, who was too fond of men and liquor.

### The difficulties with negotiating disclosure in sexual relationships

In the follow-up interviews conducted in 2008, the fears of discrimination and the difficulties in negotiating disclosure and sustaining intimate sexual relationships remained substantial themes. The most tragic account was that of Nandipha. When she needed antiretrovirals she had struggled to get access to them in the newly initiated government programme, fighting to regain her health and maintain her employment as a peer educator. She married but the relationship was conflictual. He was a drug dealer and when she was found brutally murdered, the other women were convinced that he had killed her.

Busisiwe had separated from her husband but returned to him when he contracted TB. At this point she finally disclosed that she had HIV and encouraged him to go to the clinic, as she explained in the interview: *“So*, *when I came back to stay with him again*, *I decide now that he is positive*, *I mean being sick; if he is positive he must not blame anything*, *but he must know that it has long ago been here in our home...”.* As it turned out, the reconciliation was sustained despite her revelation. Nonetheless, she had to live with the knowledge that she had felt powerless to prevent what she had feared.

Thokosizwe reported that she had not been in a sexual relationship since being left by her partner at the time of her diagnosis. She explained in the interview that she was a born again Christian and her church did not condone dating, only marriage. She was looking for an HIV-positive husband, someone she felt would be more likely to understand her situation. However, as she put it, *“I never experience a perfect relationship or a successful relationship. I used to have downfalls”.* She also spoke movingly of the insecurity she felt living alone in an area prone to violence, a fear she believed to be exacerbated by her experience years before of being raped.

Both Thandiswa and Nonkosi spoke at length of wanting to initiate and sustain relationships with men but struggling to know how to handle the matter of their positive status. The difficulties manifested at several levels: negotiating safe sex prior to disclosure; knowing at what point to disclose; knowing how to do it. Thandiswa remarked poignantly of relationships that *“I take them as if they are not serious things”*, citing the difficulties with disclosure: *“I made a mistake of disclosing to two partners. Yes*, *the difficulty is where I am going to start*, *what I am going to say. What is hard about this*, *is when do I disclose…”*. In the case of one of the partners referred to she was staying with the man and he watched her taking medication. They were using a condom, but she felt he was waiting for her to tell him. She did so when they were watching a television programme on HIV. In both instances the men professed not to have a problem with her status, but then ended the relationships because of “outside influences”. Perhaps, she reflected, they only “pretended” to understand. As a result she had changed her strategy, as she explained: *“I don’t disclose to everyone; I don’t talk I just offer a condom”*. However, even disclosing by means of a condom had not proved successful, as men did not like to use them, favouring *nyamanyama* (isiXhosa for ‘flesh to flesh’). She concluded: *“…but even there he is walking away because I have offered him a condom…I have not yet got someone who says he is staying”*.

When she was first diagnosed with HIV, Nonkosi had not been in a sexual relationship for some time, and she reflected upon how the physical manifestations of her immuno-compromised state had severely affected her self-esteem. ART had restored her health and a sustained income in HIV-related work meant that in 2008 she was economically independent and felt she had gained considerable confidence. She was proud of herself for ending an abusive relationship but was struggling to find another partner. She reflected: *“I thought my relationship was going to be easy because my partner is also HIV-positive…Now that I am a single woman*, *single mother I am looking again for a partner*, *and disclosure is my challenge. I know it has been hard for the other women because they have been rejected by men because of their status and I call that discrimination…”*. These women, from the original group involved in the book project, had told her that men simply ‘disappeared’. They had subsequently decided that they were no longer going to disclose, but just use a condom, much like Thandiswa.

Nonkosi, however, was not completely at ease with this strategy, as she described: *“I usually asked them*, *what if the person finds out*, *then what? Then they [the other women] will say that the person will probably be so in love with you and it will be hard for them to break up*, *so that is how they were defending themselves. So I was saying that*, *no it’s wrong*, *you must not do that. I mean it’s not fair on the other person”*. Her concern lay partly in the fact that men in these circumstances seldom wanted to use a condom. She continued that the other women would still justify their decision not to disclose: *“They will say that it is their [the men’s] own responsibility*, *why should I be responsible for them. If they are not responsible it is their problem”.* In addition these women added that it was also the men’s responsibility to test, suggesting that the women suspect that some men just do not want to know that they also are positive. Nonkosi went on to describe two of her recent attempts at disclosure in new relationships. In the first instance, the man professed that she was his ideal partner but that he was ready to start a family. He felt that her HIV status made it impossible for him to do so and thus to continue in the relationship. In the second instance, the man concerned said that he was too afraid of becoming infected. She noted that these men did not suggest testing themselves, being either too scared or reassured by their apparent good health. The challenge, as she put it, was to pick herself up, move on and still disclose again the next time. She was the only one of the women still involved in HIV-related work and she knew well the prevailing advice encouraging disclosure and use of a condom. Her debate about the arguments used by the other HIV positive women to justify the solutions that they were working out, reveals the complex process of negotiating understandings regarding who is responsible for protecting against infection in intimate encounters.

### The challenges of bearing a child

The women in this study largely shared the sentiments of a prevailing social discourse that values motherhood very highly for African women. When these women originally documented their experiences in the book ‘Long Life’, they displayed a considerable focus on the position of children, for example by advocating for ART on the grounds of ‘the rights of children to grow up with mothers’, and for PMTCT programmes based on ‘the rights of the child to be born negative’. In the follow-up interviews, the comments about childbearing reveal the complexity of the livelihood challenges facing these women, some related to being HIV positive, others held in common with women living in circumstances of economic fragility. The difficulties faced in providing for a child featured prominently.

Thandiswa had an unplanned pregnancy as an adolescent and at times had relied heavily on her mother to care for her daughter. During the period when she had a full-time AIDS-related income, she had turned this situation around. However in 2008 she only had part-time work and confessed in her written narrative: *“About the hope of educating my child*, *I have fear that I will not be able to because of money…”*. In 2003 Thokosizwe had written that she would not consider having a child because she was HIV-positive. However, in 2008 her position had shifted: she said that she would like a child if she could find a husband. Yet she was unemployed and in quite desperate poverty and her personal survival under these circumstances and in a crime-ridden neighbourhood appeared to be foremost in her mind.

Busisiwe and her husband only had one child, a son, whom she photographed in 2008, writing: *“[h]e is very important in my life because I got him while living with this HIV”.* She revelled in the fact that he had not contracted her HIV. Yet she also photographed her own abdomen, pregnant now for the second time, with the annotation: *“After a hard road I made a mistake of getting pregnant...I have a fear of getting sick and I am always thinking about this coming baby”*. In the interview she expressed her feeling that this recently conceived child was “bad luck”, explaining that she did not want another child when she was unemployed. Moreover, in addition to concerns about finding money to support the children, she reiterated her fear of becoming ill: *“I pray that the cure would be found by the time I get sick*, *so that I can raise my children”.* Busisiwe had been a close friend of Nandipha and reflected upon the precarious position of the latter’s two children since her death. In addition, the father of the second child had died of AIDS.

Nonkosi was the only woman who had actively sought to have a child since her diagnosis. She was delighted with her son and that he was negative *“because of ARVs”*. What is noteworthy about her experience is the motivating role played by information and support from a doctor with whom she had a longstanding therapeutic relationship. She explained: *“I did not make the decision on my own at all*, *I wanted a child but he made me decide”*. He enquired if she desired a child and then confirmed that her biological markers were such as to make it safe. As mentioned before, she had been rejected by a man who believed he was negative and thus could not have a family with her. She then ended up with a partner who was also HIV-positive but insisted that this had not been the deciding factor for her in trying to conceive at that time. She was also aware that sex with him held health risks of reinfection but she decided to go ahead with a pregnancy despite the concerns: *“I wouldn’t put that I wanted to have a child just because he is HIV-positive*, *because there was also a risk of me having an unprotected sex [with him]. I just said OK*, *let me just do it”*.

### From rights activism to regimes of responsibility

In 2002-3 these five women were involved in AIDS activism for the ‘right to health’. The right to HIV treatment was granted and the women went on to do AIDS-related work. By 2008 this was only the case for Nonkosi. The loss of this income had affected the other women’s general wellbeing significantly. In addition, they had lost their entitlement to state disability grants in an exercise of rationalisation of benefits, in particular for chronic illness. There was a strong sense that they had returned to facing the same struggles of poverty and unemployment that they recognised from prior to their HIV diagnosis, a predicament shared with many other women living in low-income areas in Cape Town. At the same time ART had restored their physical health, making their illness less visible. As Thandiswa commented: *“...now I know it’s still present but I didn’t see myself as having it”.* The availability of treatment had reduced having HIV to a more private, individualised experience. None of the women attended support groups any longer; they were *“living their own lives*, *with their own HIV”*, as Nonkosi put it. However, she acknowledged that the appearance of being like other people was only possible if one maintained strict adherence to a drug regimen, a paradox she captured as follows: *“[s]ometimes I forget that I have HIV anyway*, *I am just taking this medication”.* The women expressed a strong sense of personal responsibility to remember the pills, to live healthily, to attend appointments and collect medication.

The interviews indicate that they were now inclined to interpret their bodily states in terms of biological markers, so central to managing HIV as a chronic illness. Indeed it was these markers that enabled clinic staff to check if they were conforming. The descriptions of interactions in the clinic indicate a concern not to end up in a situation of being “punished” for non-adherence, by having to endure more frequent appointments and home visits. In addition, the women emphasised a personal commitment to heeding the warnings of health professionals not to end up on second-line therapy, or contract drug-resistant infections. As the language of rights was so central to the book, the language of personal responsibility had become prominent in their lives. Behaving responsibly was seen by them as key to staying well, and their memories of illness easily summoned a motivating fear. Busisiwe acknowledged that there were still HIV-positive people who became very ill, yet added: *“[b]ut I also think that people who are like that are people who choose to be like that…because people mix alcohol and ARVs or a person forgets to take them or spits them when they are sick…”*. Thus the women could also judge those not conforming to the rules of the regime, as much as they were still defining for themselves where the boundaries of personal responsibility lay in their own private lives.

## Discussion

The HIV positive women whose perspectives on their sexual and reproductive lives are described above have personally experienced the benefit of gaining access to ART through advocacy efforts asserting their individual rights to health. Apart from a role as activists campaigning for rights, they have been ‘experts’ educating others about living with HIV, and are themselves also patients subject to the surveillance of treatment regimes. The majority of them are Christians who consider the support of their churches central to coping with chronic illness. Furthermore, they are part of communities where strong social discourses prevail regarding HIV, childbearing and gender hierarchies. Clearly all these experiences will have influenced their understandings of their rights as women and as citizens, and also their notions of responsibilities. It is evident from the follow-up study, however, that they faced several constraints in fully actualising their SRH rights and some rights had greater relevance to their circumstances than others. The impression gained from the descriptions of their lives in 2008 is that several SRH rights remained an ideal as opposed to a reality. Having lost certain income benefits that came in the early years of having HIV, most of them were again struggling to maintain a basic level of economic survival, alongside other realities that compromised their broader wellbeing. It is thus understandable that HIV activist organisations have expanded their emphasis also to advocating for broader socio-economic rights.

It is evident from the follow-up interviews that the women’s experience of living with HIV had also become more private and focused on responsibilities, reflected particularly in a sense of responsibility personally to remain healthy and adhere to medication, as espoused in treatment programmes. However, negotiating the balance between the reality of their circumstances and directives about the responsibilities ensuing from a positive status, turned out to be a complex matter in practice. These difficulties become most salient in the dilemmas expressed with respect to disclosure. It is apparent that the women cherished desires for fulfilling sexual relationships, but these were hard to achieve in the face of the considerable personal risks associated with revealing HIV status. The clinic directive to practise safe sex with condoms to prevent infecting others (and indeed re-infection of yourself) was understood to be an ideal. It inferred a responsibility to protect a sexual partner. However, in practice it had to be reworked, as these women most often lacked the power in intimate relationships to insist on the use of condoms. They felt unable at times to pursue a policy of upfront disclosure of their status due to fear of stigma, abandonment, loss of financial support and even violence. To the existing inequality in gender relations would be added the factor of their positive status in a seemingly discordant relationship. Some women thus resorted to a compromise in the non-verbal: the offer of a condom was still referred to as ‘disclosure’.

The twist on the responsibility discourse of the clinic that enabled justification of this compromise, erased responsibility for the other by implying that each person bears responsibility largely to protect him/herself. Such a justification might be rationalised into an extension of the emphasis at the clinic on individuals becoming responsible for their own health. Thus it is also a man’s responsibility to protect himself and indeed to find out his own status. In many ways it is understandable how women in such settings might feel that with respect to their sexual encounters, they can only be responsible for themselves. Even if they do feel some responsibility for the sexual partner, as Busisiwe did, they are often either limited in the extent they feel able to act on that sense, or doing so carries the risk of personal repercussions. Not disclosing and at times having unsafe sex is then justified with the assertion that men must also take responsibility for their health and sexual safety and use condoms and testing services. Of course there are risks of a new HIV or other infection for the women if the man turns down the offer of the condom, but perhaps at times these risks appear less significant than others immediately in their life path. Women living in these circumstances have to negotiate the extent to which they can take responsibility for others and they justify the instances where this is not possible. The right that they found most salient in such intimate encounters appears to be a right to keep their status confidential, to maintain silence.

The women’s concerns around disclosure point to a complex field [33] in which socio-economically underprivileged women negotiate their relationships, with medicine, health professionals and with their partners. Further, a rights-based approach to HIV is conceptually nebulous [20,21,23,26,34], and although it is crucial for international and national bodies to accord defined rights to citizens, the realisation of rights is problematic in contexts such as South Africa with a plural legal system and an over-burdened justice system [20,22,35]. Consideration of the South African example adds a further dimension to debates about contextualising rights by illustrating very real political tensions between universal notions of human rights and more relativist positions. The existence of a plural legal framework, which includes customary law, creates a contested domain encapsulating sometimes contradictory legal references. Arguments problematise an imposition of ‘international’ (and implicitly ‘western’) conceptions of the individual that then supersede historical (and constructed ‘African’) legacies of the communal [35]. Thus, in addition to granting individuals the extensive set of rights set out in the International Bill of Human Rights, the South African legal structure specifically recognises collective rights, for example in the form of the Recognition of Customary Marriages Act (1999). This act condones the so-called ‘African cultural practice’ of marriage in which women fall under the guardianship of their husbands and are considered to be legal minors.

In considering the realisation of rights, and the corresponding sets of responsibilities attached to rights in the context of South Africa, we argue that it is critical to understand the ways in which agency is constrained, in particular by gender inequality [16,36]. The discourse of responsibility advocated by health centres, especially through ART programmes, accords both agency and responsibility to individual women, many of whom have entered the biomedical system and tested for HIV when they became pregnant. The broader context in which women live is also considered important to health professionals, but largely to the extent that the women are encouraged to disclose to one person – a supporter – prior to commencing ARVs. Based on the women’s accounts, they both adopt and resist this discourse, pointing also to the disciplining aspects of a discourse of ‘responsibilised’ health citizenship [37]. Individual responsibilities linked to rights, like the right to privacy and the right to access treatment, may not extend to an individual’s sense of responsibility to the collective, through adopting practices that prevent HIV transmission but which may also violate their right to privacy. The narratives from the women suggest that they are reframing the ‘burden’ of the responsibility to disclose and ‘protect’ their partner. Instead, they maintain their silence in order to ‘protect’ themselves from discrimination while offering condoms for protection. On the other hand, the women’s accounts reflect their commitment to ensuring their own wellbeing through self-care, including healthy eating practices. Women, we suggest, adopt tactics within a context characterised by various inequalities in order to ‘make do’ [24]. The extent to which they can realise the rights that are available to them on paper is limited by their ability to negotiate their socio-economic circumstances, and their relationships with their partners and with health care professionals in particular.

Other studies have found similar dynamics, with women expressing a determination and desire to have safe sex while feeling constrained in their ability to insist, citing the same fears around disclosure as the women whose experiences we have detailed [28,38-40]. In a series of studies attached to a PMTCT initiative [41] in Abidjan, Ivory Coast, 149 HIV-positive women thought to be in ‘stable’ partnerships were followed up post-partum. In a survey, only 40% of these women had told their partners that they were infected with HIV. Amongst those who were sexually active, and had not disclosed, 76% reported never using condoms. The author concludes that non-disclosure was largely motivated by a fear that the man would leave them, with severe economic consequences.

Section 27, a South African legal rights organisation, points to fears of sexual violence and abandonment as key in preventing women from disclosing [44] and echoes the UNAIDS approach in challenging criminalisation of HIV transmission. There is also evidence that prevailing societal attitudes in South Africa do not make personal decisions about how to enjoy a sex life as an HIV positive woman any easier. A survey [42] that assessed popular attitudes to sexual activity by HIV-positive people found that amongst a sample of all women attending general primary care clinics in the Western Cape, 46% believed that HIV positive people should not remain sexually active. In the same survey, 77% of women felt that HIV-infected individuals should not have children [42]. This is in a context where involuntary childlessness in itself carries significant stigma [45]. A cross-sectional survey of fertility intentions amongst 459 men and women living with HIV in black urban and peri-urban working class communities in Cape Town [46], found that forty-five percent of women were open to the possibility of having a child. However, a comparatively small proportion of women and men in this survey had discussed reproductive options with a health professional.

Health care workers’ attitudes and the degree of information offered in clinics are important factors in women’s choices regarding childbearing [7,28,29]. A qualitative study with newly diagnosed HIV-positive women found that concerns regarding the dangers of childbearing (such as vertical transmission) are mitigated by discussion of their fertility options [28]. The attitudes of health care providers in South Africa are reportedly dominated by medical concerns related to the risks of pregnancy for the HIV-positive women [29] and the provision of contraception within ART programmes has received more emphasis than services promoting reproductive choice [7]. In this way, a focus on access to contraception to the exclusion of offering information can in fact inadvertently synchronise with negative attitudes and contribute to reducing the ability of HIV-positive women to have an informed choice with respect to other options. This could be seen to infringe their right to autonomous decision-making. London et al [47] argue that the current non-prescriptive nature of guidelines regarding HIV and reproduction in South Africa increases the risk of the views of health workers influencing how a person’s right to choice is negotiated in a clinic interaction.

## Conclusions

Individual autonomy features in legal guidelines regarding both sexual activity and childbearing for HIV-positive people. Yet responsibility is also foregrounded, albeit as an ‘ethical’ matter as opposed to a legal imperative, and is mirrored in the language of responsibility that has entered into treatment regimes. There is a danger that this language of responsibility can come to bolster more pervasive moral discourses and societal notions of blame that can make the environment in which HIV-positive women have to negotiate their sexuality and fertility more fraught. Notions of responsibility can translate on the ground into moralistic directives that intrude into clinic spaces and have unintended effects. Interactions with health care workers may accentuate existing feelings of guilt and undermine necessary strategies that women have employed to negotiate HIV infection in their day-to-day lives. Even in contexts like South Africa, where rights have been prominent in HIV programming, these framings of ‘responsibilities’ can in fact encourage practices on the part of health care workers that diverge from an approach respecting individual rights, such as the right to choice. This might occur, for example, when options regarding pregnancy are not presented to HIV-positive women or supported by their health professionals. Pressure to disclose status can inhibit rights that have meaning for women, such as a right to silence. This situation is compounded by the reality that antenatal testing has contributed to a gendered profile with respect to the uptake of testing and treatment in South Africa. In the application of SRH policy in the public sector and in the minds of health personnel, women can come to be invested with a greater responsibility to disclose and prevent transmission of the virus, in part because men are not as frequently drawn into the biomedical health system.

It has been argued that anthropological analyses of human rights contribute to critical approaches “…insofar as they stress human sociality as opposed to the foundation for human rights found in conventional liberal accounts” ([50]. p7). This point is borne out in Ross’ account [34] of women who applied to testify in the Truth and Reconciliation Commission in South Africa. She contends that the legal language attendant upon universal human rights discourses can ignore the complexity of shifting webs of social relations within which discussions of rights necessarily come to be embedded. The findings discussed in this paper would suggest that a nuanced interpretation of the social fabric within which legal rights and the attendant responsibilities are translated and implemented is necessary to appreciate the particular position of women and the ways in which interpretations of moral agency come to be played out in different social spaces. This is especially so given that the gendered nature of testing uptake contributes to conceptualisations of women as especially responsible for reducing transmission. Of further salience is the fact that a situation persists where gender inequality contributes to a high HIV prevalence and where customary laws, that can further reinforce women’s unequal position, occupy an uneasy position alongside universal concepts of human rights.

## List of abbreviations used

AIDS: Acquired Immune Deficiency Syndrome; ANC: African National Congress; ART: Antiretroviral Therapy; ARV: Antiretroviral; HAART: Highly Active Antiretroviral Therapy; HIV: Human Immunodeficiency Virus; MSF: Médicins sans Frontières; PMTCT: Prevention of Mother to Child Transmission; SRH: Sexual and Reproductive Health; TAC: Treatment Action Campaign; UNAIDS: Joint United Nations Programme on HIV/AIDS.

## Competing interests

The authors declare that they have no competing interests.

## Authors’ contributions

HM wrote the first draft of the paper and coordinated revisions. Both authors contributed to the analysis, subsequent drafts and conclusions.
